# Application of Thin ZnO ALD Layers in Fiber-Optic Fabry-Pérot Sensing Interferometers

**DOI:** 10.3390/s16030416

**Published:** 2016-03-22

**Authors:** Daria Majchrowicz, Marzena Hirsch, Paweł Wierzba, Michael Bechelany, Roman Viter, Małgorzata Jędrzejewska‑Szczerska

**Affiliations:** 1 Department of Metrology and Optoelectronics, Faculty of Electronics, Telecommunications and Informatics, Gdańsk University of Technology, Narutowicza Street 11/12, Gdańsk 80-233, Poland; majchrowiczdaria@gmail.com (D.M.); plutuska@gmail.com (M.H.); pwierzba@eti.pg.gda.pl (P.W.); 2 Institut Européen des Membranes, UMR-5635, Université de Montpellier, The École Nationale Supérieure de Chimie de Montpellier, Centre national de la recherche scientifique, Place Eugène Bataillon, Montpellier 34095, France; mbechela@univ-montp2.fr; 3 Institute of Atomic Physics and Spectroscopy, University of Latvia, 19 Raina Blvd., Riga LV-1586, Latvia; viter_r@mail.ru

**Keywords:** Fabry-Pérot interferometer, Atomic Layer Deposition, ZnO layer, interference

## Abstract

In this paper we investigated the response of a fiber-optic Fabry-Pérot sensing interferometer with thin ZnO layers deposited on the end faces of the optical fibers forming the cavity. Standard telecommunication single-mode optical fiber (SMF-28) segments were used with the thin ZnO layers deposited by Atomic Layer Deposition (ALD). Measurements were performed with the interferometer illuminated by two broadband sources operating at 1300 nm and 1550 nm. Reflected interference signal was acquired by an optical spectrum analyzer while the length of the air cavity was varied. Thickness of the ZnO layers used in the experiments was 50 nm, 100 nm, and 200 nm. Uncoated SMF-28 fiber was also used as a reference. Based on the results of measurements, the thickness of the ZnO layers and the length of the cavity were selected in order to achieve good visibility. Following, the interferometer was used to determine the refractive index of selected liquids.

## 1. Introduction

Fiber-optic Fabry-Pérot interferometric sensors are one of the most widespread types of fiber-optic sensors. They are widely used in measuring physical quantities, such as temperature, pressure, refractive index and humidity, as well as in chemical and biological sensing, e.g., to determine sugar content in aqueous solutions [1,2,3,4,5,6,7,8,9] or hematocrit level in human blood [10,11,12,13]. Their main advantages—high sensitivity and immunity to mechanical disturbances and to electromagnetic interference (EMI), combined with their compact size and light weight, stimulate the research into new and improved types of such sensors. This research benefits from the progress in material science and photonics, providing new materials (e.g., diamond) and manufacturing technologies, such as Atomic Layer Deposition (ALD).

Zinc oxide (ZnO) is a promising wide band-gap semiconductor material with many interesting properties, *i.e.*, high transparency in visible light, tunable electrical conductivity and piezoelectric properties [14]. Most of the research on using ZnO focuses on applications in optoelectronic devices such as LEDs [15,16], a light-driven optofluidic switch [17], optoelectronic modulators [18] or solar cells [19] as well as on its applications in electric sensors of gas or humidity [20,21,22].

Among the techniques which are used for the synthesis of thin ZnO layers, Atomic Layer Deposition (ALD) [14] has important advantages for applications in optoelectronics. Using controlled deposition of single-atomic layers, thin films are grown, exhibiting little variation in the thickness. Structural properties, like grain size or surface morphology, can be changed by adjusting the parameters of the deposition process [5]. While the low growth rate in ALD technique limits the maximum practical thickness of synthesised layers to below half a micrometer [20], such layers can be used in fiber-optic Fabry-Pérot interferometers to modify the properties of their reflective layers.

**Figure 1:**
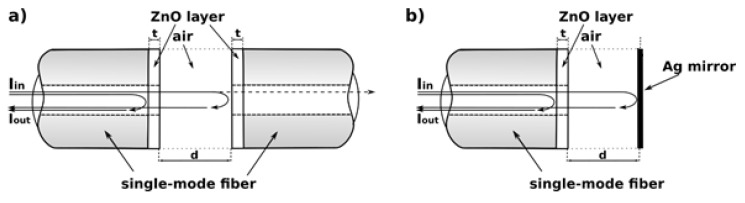
The construction of the fiber-optic Fabry-Pérot interferometer in the reflective mode: (**a**) all-fiber (with both interferometer mirrors made of ZnO layer); (**b**) with one of the interferometer mirrors in the form of a silver plate, where: d—the cavity length, t—thickness of the ZnO layer.

The purpose of this paper is to study metrological parameters of the fiber-optic Fabry-Pérot interferometer with thin ZnO layers deposited using ALD on the end faces of the single-mode optical fibers which form the Fabry-Pérot cavity. Several interferometers were manufactured from a standard telecommunication single-mode SMF-28 fiber. They have been operating in the reflection mode. The construction of such an interferometer is shown schematically in Figure 1. The thickness of the deposited ZnO layers used in the study was 50 nm, 100 nm and 200 nm. Additionally, a Fabry-Pérot interferometer without the ZnO coating layers was tested, acting as the reference.

## 2. Materials and Methods

### 2.1. ZnO Thin Layers

The investigated Fabry-Pérot interferometer was built of two standard telecommunication single-mode SMF-28 optical fibers (Thorlabs, Inc., Newton, NJ, USA) with thin ZnO layers grown on the end-faces, positioned as shown in Figure 1, with an air cavity. In this study, the ZnO layers of equal thickness of 50 nm, 100 nm and 200 nm were used for both fibers. The layers were deposited by ALD using a custom made ALD reactor [5,23,24]. Layer deposition was achieved using sequential exposures of diethylzinc (DEZ) and H_2_O separated by a purge of Argon with a flow rate of 100 sccm. The deposition regime for ZnO consisted of 0.1 s pulse of DEZ, 20 s of exposure to DEZ, 40 s of purge with argon and 2 s pulse of H_2_O, 30 s of exposure to H_2_O and finally 60 s purge with argon. A total of 250, 500 and 1000 cycles were performed on the optical fibers at 100 °C in order to obtain 50, 100 and 200 nm thick ZnO respectively. The ALD ZnO layers used in this study have been previously characterized and the results were published elsewhere [5,23,24].

### 2.2. Fabry-Pérot Interferometer

The investigated Fabry-Pérot interferometer, shown in Figure 1, can be considered as a triple cavity interferometer, with side cavities formed by the ZnO layers and the main cavity located between these layers. Calculation of the transmission or the reflection of such an interferometer, especially when illuminated by a near-Gaussian beam from a single-mode fiber, is a computationally intensive task.

However, as the thickness of the ZnO layers is always below 1/5th of operating wavelength, it is safe to assume that the diameter of the near-Gaussian beam illuminating such layer does not expand significantly upon reflection or transmission. Therefore, the ZnO layers can be approximated by reflective surfaces whose reflection and transmission is assumed equal to the reflection and transmission of the Fabry-Pérot interferometer formed by the ZnO layer placed between the optical fiber and the main cavity, and illuminated by a plane wave.

Assuming that the ZnO layers do not exhibit birefringence and the refractive index of ZnO layer is higher than that of the surrounding media, reflectivity *R*_1_ of the boundary between the optical fiber (refractive index n_1_) and the ZnO layer (refractive index n_ZnO_) and reflectivity *R*_2_ of the boundary between the material of the cavity and the ZnO layer can be expressed as:

(1) $$R_{1}=\frac{n_{ZnO}-n_{1}}{n_{1}+n_{ZnO}}$$

(2) $$R_{2}=\frac{n_{2}-n_{ZnO}}{n_{ZnO}+n_{2}}$$

The phase difference introduced in the ZnO layer is given by:

(3) $$\delta =\frac{2\pi }{\lambda }tn_{ZnO}$$

where: *λ*—wavelength, *t*—thickness of the ZnO layer, *n_ZnO_*—refractive index of ZnO layer. Therefore, the reflectivity ℜ of the ZnO layer can be expressed as:

(4) $$ℜ=\frac{R_{1}+R_{2}-2\sqrt{R_{1}R_{2}}cos\delta }{1+R_{1}R_{2}-2\sqrt{R_{1}R_{2}}cos\delta }$$

The refractive index of the ZnO layer can be conveniently calculated from [19]:

(5) $$n_{ZnO}^{2}=2.81418+\frac{0.87968\lambda^{2}}{\lambda^{2}-{\left(0.3042\right)}^{2}}-0.0711\lambda^{2}$$

where *λ*—wavelength expressed in µm.

**Figure 2:**
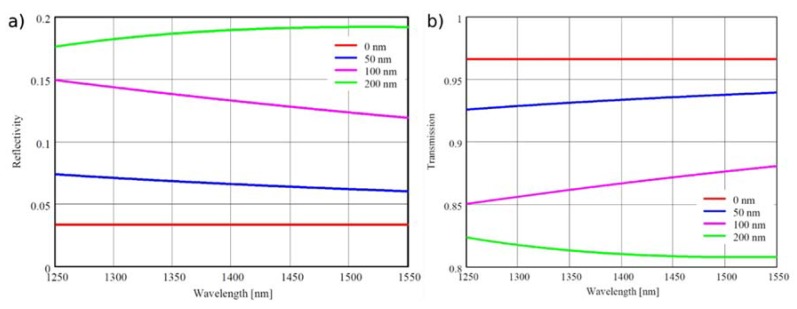
Calculated reflectivity (**a**) and transmissivity (**b**) of a 50 nm, 100 nm and 200 nm ZnO layer on the end face of an SMF-28 fiber. Reflectivity and transmission of an uncoated SMF-28 fiber end face shown for comparison fiber.

Equations (1)–(5) were used for calculating the reflectivity ℜ and transmissivity (1 − ℜ) of the ZnO layers in the spectral range from 1250 nm to 1550 nm. The results of calculations are presented in Figure 2 for ZnO layers of thickness 50 nm, 100 nm and 200 nm (Figure 2a,b—reflectivity and transmissivity of layer, respectively). The layers of 200 nm thickness were chosen because of their relatively high value of reflectivity in the wavelength in the range of 1300–1550 nm.

The relatively high value of reflectivity of the 200 nm ZnO layer is related to interference of the optical radiation reflected from both the surfaces of the layer. As the optical path difference between interfering waves is the closest to a wavelength for the 200 nm ZnO layer, the amplitudes of these waves add, rather than subtract, leading to a high value of reflectivity. The reflectivity does not depend on the crystalline properties of the ZnO layer or on a band-to-band transition.

The investigated Fabry-Pérot interferometer was modelled as the main cavity delimited by plane surfaces whose reflectivity was ℜ (given by Equation (4)), illuminated by a Gaussian beam from the single-mode fiber. Reflectivity spectra of this interferometer were computed for different cavity lengths and thickness of ZnO layers and the interference contrast *V* was calculated from [25,26]:

(6) $$V=\frac{I_{\max}-I_{\min}}{I_{\max}+I_{\min}}$$

where: *I_max_* is the maximum intensity of the measured spectra, *I*_min_ is the minimum intensity of the measured spectra.

From the results of these calculations, it follows that the cavity length should be in the range from 50 µm to 200 µm in order to ensure optimal operation of the investigated interferometer.

### 2.3. Measurement Set-Up

**Figure 3:**
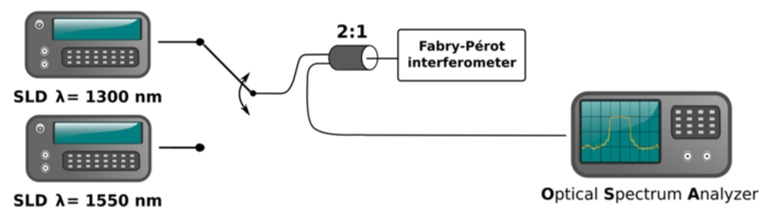
Measurements setup for fiber-optic Fabry-Pérot interferometer in reflection mode.

In order to study the response of the fiber-optic Fabry-Pérot interferometer, the measurement setup shown in Figure 3 was built. It consists of a broadband source, the investigated Fabry-Pérot interferometer operating in reflection mode and an optical spectrum analyser used for as a detector.

**Figure 4:**
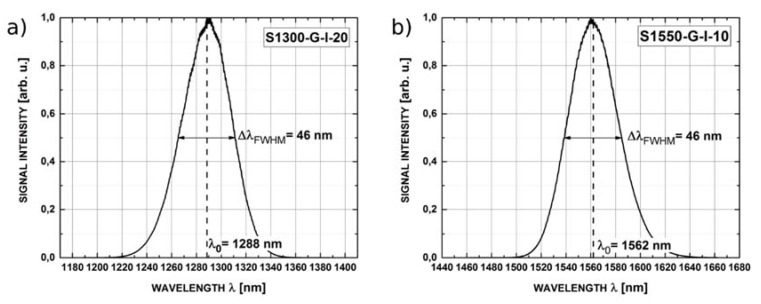
Intensity for implemented superluminescent diodes with marked center wavelength (λ_0_) and full width at half maximum (Δλ_FWHM_) for: (**a**) S1300-G-I-20; (**b**) S1550-G-I-10.

Two broadband sources based on superluminescent diodes, S1300-G-I-20 and S1550-G-I-10 (Superlum Ltd., Carrigtohill, Ireland) were used in the setup. Their central wavelengths were 1288 nm and 1562 nm, respectively. Spectral width of both sources, measured as full width at half maximum (FWHM), was about 50 nm, as shown in Figure 4.

Detection of the signal from the investigated Fabry-Pérot interferometer was performed by an Ando AQ6319 optical spectrum analyzer (Yokogawa, Tokyo, Japan). All optical fiber connections in the setup were made using single-mode SMF-28 fiber (Thorlabs, Inc., Newton, NJ, USA).

Measurements were always conducted with only one source connected to the setup, in order to reduce the power level in the fibers, and minimize the amount of power rejected by the optical spectrum analyzer; the connection was performed manually, prior to the measurement. Resolution bandwidth of the optical spectrum analyzer was set to acquire at least ten samples per expected fringe period, mostly from 0.1 nm to 1.0 nm. For each source, a series of measurements were performed for cavity length stepped from 0 μm to 500 μm in increments of 50 μm.

## 3. Results and Discussion

**Figure 5:**
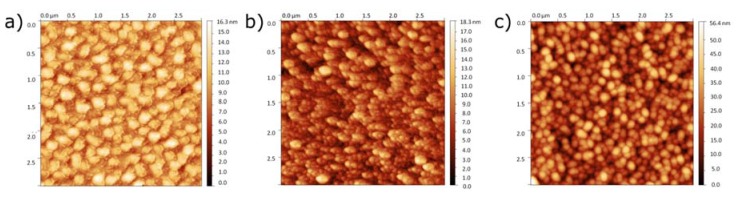
AFM image of ALD ZnO nanolayers with difference thickness: (**a**) 50 nm; (**b**) 100 nm and (**c**) 200 nm.

Previously, we have reported on optical and structure properties of ALD ZnO nanolayers [5,23,27]. We revealed the polycrystalline structure of 200 nm ZnO layers [28]. The fabricated ZnO layers demonstrate a rough surface with columnar growth and an average size of the grains in the range of 5–60 nm as a function of ZnO film thickness (Figure 5).

The surface morphology was studied by an Asylum Research MFP-3D Atomic Force Microscope, operating in tapping mode and equipped with a commercial silicon tip. The obtained image was analyzed using the free software Gwyddion. From the AFM image (Figure 5a) it is clearly seen that ZnO nanolayers of 50 nm thickness were amorphous. In this case, agglomerates of 100–200 nm were found on the surface. On the other hand, ZnO crystallization has been improved for 100 nm layers (Figure 5b), so the agglomerates with well defined shapes of 20–40 nm have been observed (Figure 5b). ZnO nanolayers of 200 nm showed good crystalline structure with agglomerates of 50–150 nm (Figure 5c). From Figure 5, one can see that the surface roughness of the ZnO layers increased with the thickness enhance.

Previously, with the use of XRD technique we have shown that surface agglomerates of ALD ZnO nanolayers consisted of smaller grains of 7–15 nm in diameter [28]. It was also shown that thicker ZnO films showed a higher band gap and a lower defect concentration due to a higher crystallinity [28]. All this is important for applications in optoelectronics in IR and mid-IR regions in which the absorption on defects, free electrons and scattering effects are significant [21,29,30].

**Figure 6:**
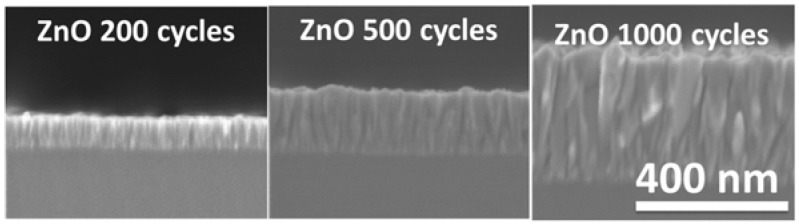
SEM image of ALD ZnO nanolayers with 200, 500 and 1000 cycles.

A scanning electron microscope with a tungsten source and variable chamber pressure (VP-SEM) was used to define the ZnO layers. Figure 6 shows SEM micrographs of ALD ZnO nanolayers with 200, 500 and 1000 cycles. The continuity and the complete coverage of the layers can be observed. The 200 nm ZnO layers were transparent in the range of wavelengths from 440 to 1100 nm [23,28]. The absorption edge of the ZnO layers was observed between 340 and 440 nm which is typical of ZnO nanostructures deposited by ALD. The band gap of the ZnO layers was estimated to be 3.28 eV [23,28].

**Figure 7:**
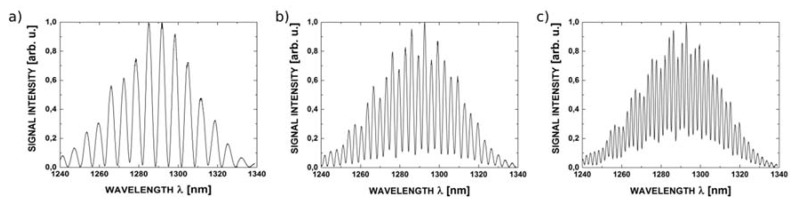
The measurement signal with the central wavelength 1300 nm for ZnO layer thickness of 200 nm and the length of Fabry-Pérot cavity: (**a**) 100 μm; (**b**) 200 μm and (**c**) 300 μm.

**Figure 8:**
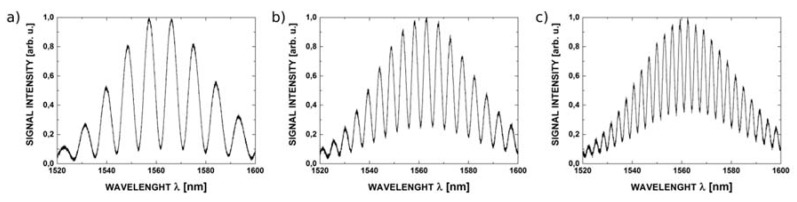
The measurement signal with the central wavelength 1550 nm for ZnO layer thickness of 200 nm and the length of Fabry-Pérot cavity: (**a**) 100 μm; (**b**) 200 μm and (**c**) 300 μm.

The reflectance spectra obtained during the measurements feature good quality interference, providing a detailed insight into the performance of the investigated interferometer. Example spectra acquired for cavity length from 100 μm to 300 μm with broadband source operating at 1300 nm are shown in Figure 7, while those acquired with the 1550 nm source are presented in Figure 8.

In the identification of the optimal length range of the Fabry-Pérot cavity, two factors had to be taken into account: the interference contrast *V* and the spacing between the fringes (*i.e.*, the free spectral range). While *V* close to one is desirable, the accuracy of virtually all detection setups does not degrade significantly with *V* decreasing to 0.8 or even 0.7. The spacing between the fringes should be adjusted in such a way that the spectrum contains enough fringes for selected processing algorithm (mostly about eight to ten) but the fringes are not too closely spaced.

In most cases increasing the number of fringes in the spectrum above a certain level does not improve the performance of the sensor, while requiring a high-resolution spectral detection and increasing the amount of data to be processed.

**Figure 9:**
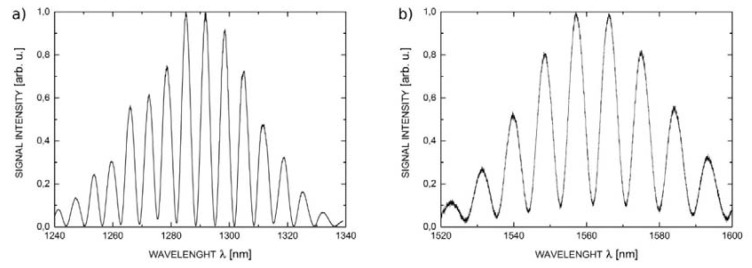
Representative measured spectra from Fabry-Pérot interferometer with 200 nm ZnO layers (cavity dimension: 100 μm) for source wavelengths: (**a**) 1300 nm; (**b**) 1550 nm.

**Figure 10:**
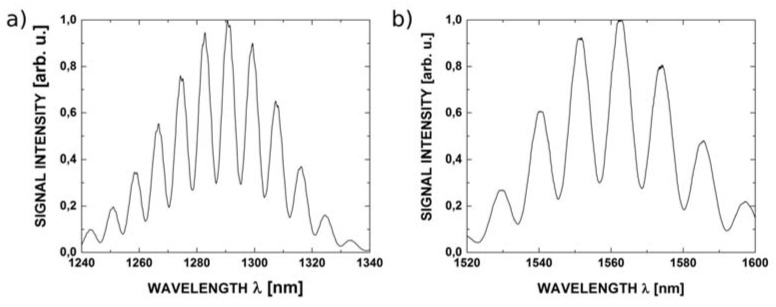
Representative measured spectra from Fabry-Pérot interferometer with 200 nm ZnO layers (cavity dimension: 100 μm) for source wavelengths: (**a**) 1300 nm; (**b**) 1550 nm.

**Figure 11:**
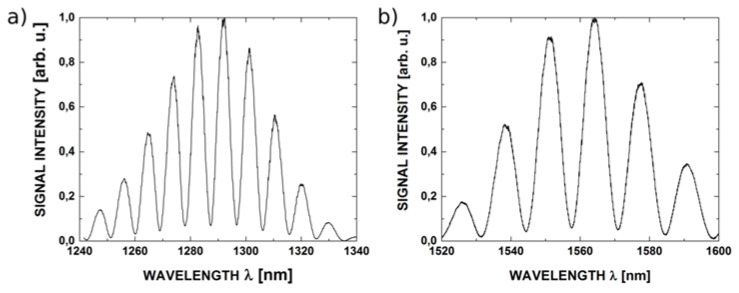
Measured spectra for 50 nm ZnO layers (cavity dimension: 100 μm) for best visibility of the fringes and signal intensity at wavelength (**a**) 1300 nm; (**b**) 1550 nm.

The measured spectra from the set up made with the use of fiber with ZnO layers of 50, 100, 200 nm respectively are presented below. From Figure 9, Figure 10 and Figure 11, it can be noted, the highest value of visibility is offered by the set up with the 200 nm ZnO layers. The high value of visibility gives us opportunity to obtain the best metrological parameters of the sensors.

Taking into consideration the above discussion and the results of measurements, it can be stated that optimal length of the cavity of the investigated Fabry-Pérot interferometer with 200 nm ZnO layers is around 100 μm. The measured spectra for such an interferometer, acquired with the 1300 nm source and 1550 nm source, are presented in Figure 9a,b, respectively.

Interference contrast *V* for the spectrum acquired at 1300 nm was 0.964 and 0.818 for that acquired at 1550 nm. Such high values of *V* indicate that no significant changes in the state of polarization of the interfering beams take place. Otherwise, the contrast would have been substantially reduced.

To measure the refractive index of the chemical liquids, the test sample was placed between the tip of the optical fiber and a mirror mounted on an adjustable table. To obtain a measurement result, spectral separation should be read from measured signal spectrum and compared with reference spectrum. It allows to obtain the relationship between the distance between the position of adjacent maximums in the measured signal spectrum and the refractive index of the sample.

**Figure 12:**
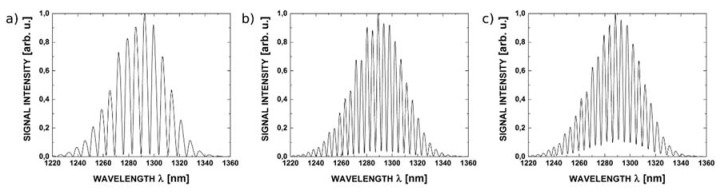
Measurements of different chemical substances, Fabry-Pérot cavity length 100 μm, at wavelength 1300 nm: (**a**) reference for clean Ag mirror; (**b**) cyclohexane and (**c**) toluene.

**Figure 13:**
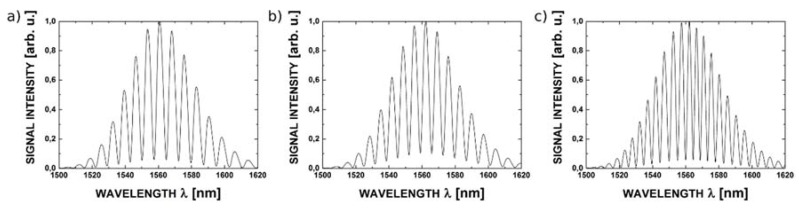
Measurements of different chemical substances, Fabry-Pérot cavity length of 100 μm, at wavelength of 1550 nm: (**a**) reference for clean Ag mirror; (**b**) cyclohexane and (**c**) toluene.

In the second part of the study the investigated Fabry-Pérot interferometer with 200 nm ZnO layers, the cavity length of 100 μm, operating in the reflective mode, was used to measure the refractive index (n) of selected chemical substances: cyclohexane, benzene, toluene, issue oil, paraffin. The spectra corresponding to these substances measured with the use of the sources with central wavelength 1300 nm and 1550 nm are presented in Figure 12 and Figure 13, respectively.

It can be noted from Figure 12 and Figure 13 that the spectral modulation of the measured signal varies with the change of refractive index of liquid which filled the Fabry-Pérot cavity. The determined optimal Fabry-Pérot cavity length is large enough that a broad range of liquid and solid substances can be used to fill the cavity.

High value of visibility achieved in the presented setup shows also a potential for future work where the applications of ZnO extend beyond a reflective layer. It is also worthwhile to note here that ZnO properties can be tuned easily by ALD, if needed, as it has been demonstrated elsewhere [23,27,28].

The average refractive index was calculated for all the substances for the wavelength range 1260 nm–1320 nm and 1530 nm–1590 nm. Calculations of refractive index values were performed using a method described in [4]. In this method the measured spectrum is divided by the spectrum of the source and corrected for spectral characteristics of the cavity mirrors, if necessary. Following, the product of the cavity length and an average refractive index is obtained from the locations of minima and maxima in the processed measurement spectrum. Finally, knowing the length of the cavity, the refractive index of the test sample is calculated). The results of calculations are presented in Table 1, with reference values of the refractive indices for comparison. It can be seen that our sensor has the ability to measure refractive index with appropriate metrological parameters.

One of the most important parameters of every sensor is its measurement uncertainty. In order to assess the uncertainty of our setup, a simple model was used. In this model it was assumed that the length of the cavity h and the refractive index n are determined by a fairly simple method, in which wavelengths *λ_1_* and *λ_2_* corresponding to two maxima or minima on the processed measurement spectrum are used for calculations. It was also assumed that the *λ_2_* − *λ_1_* = 60 nm and that *λ_1_* and *λ_2_* are known with uncertainty of 0.01 nm. Writing:

$$\frac{\Delta h}{h}=\frac{1}{h}\left|\frac{\partial h}{\partial \lambda_{1}}\Delta \lambda_{1}\right|+\frac{1}{h}\left|\frac{\partial h}{\partial \lambda_{2}}\Delta \lambda_{2}\right|$$

We obtain *Δh/h* = 3.3 × 10^−4^. Similarly, writing:

$$\frac{\Delta n}{n}=\frac{1}{n}\left|\frac{\partial n}{\partial \lambda_{1}}\Delta \lambda_{1}\right|+\frac{1}{n}\left|\frac{\partial n}{\partial \lambda_{2}}\Delta \lambda_{2}\right|+\frac{1}{n}\left|\frac{\partial n}{\partial h}\Delta h\right|$$

Δ*n/n* = 6.6 × 10^−4^, which gives Δ*n* ≈ 0.001 for *n* around 1.5. Such an uncertainty is sufficient for several applications. It can be further reduced by reducing the uncertainty of wavelength measurement and improving the spectral stability of the source or using a method that determines *h* and *n* based on the entire processed measurement spectrum, rather than on two points.

Hysteresis and drift are effects that often degrade the performance of a sensor. In order to determine, if they are present in the investigated setup over 300 measurements were performed. For each measurement a spectrum was recorded for the sensor with the empty cavity. Following, the cavity was filled with the liquid to be measured, and the second spectrum was recorded. The liquid was removed from the cavity and isopropyl alcohol was used as the cleaning agent. Finally, when the alcohol has evaporated, the third spectrum was recorded. Comparing the first and the third spectra, it can be concluded that no hysteresis or drift is present. Similarly, different spectra corresponding to a given liquid are virtually identical, indicating that the repeatability of the measurements is very high. Lack of hysteresis and drift in the presented setup is most probably the result of using pure chemicals, a short measurement time and cleaning with isopropyl alcohol after each measurement. After all the measurements had been performed, the ZnO layer was inspected using optical microscopy in order to assess its quality and detect any signs of damage caused by the chemicals.

## 4. Conclusions

In the presented study, the response of a fiber-optic Fabry-Pérot interferometer with thin ZnO layers deposited using ALD on the end faces of the single-mode optical fibers forming the cavity was studied. Performance of this interferometer operating in the reflective mode was examined at two NIR wavelengths (1300 nm and 1550 nm). The investigated Fabry-Pérot interferometer showed good values of the interference contrast *V*, while the ZnO layers did not degrade the state of polarization of interfering beams. Optimal cavity length (100 µm) was determined as a compromise between good interference contrast and the number of fringes in the acquired spectrum. Following, the investigated interferometer in its optimal configuration was used to measure refractive index of selected chemical substances. The obtained results confirmed the correct operation of the interferometer and demonstrated its capacity for successful integration in sensing applications. The application scope of this sensor can be further extended by applying a protective coating, such as diamond, on the ZnO layers.

## Figures and Tables

**Table 1:** Representative measured and reference values of the refractive index for investigated substances.

Name	Wavelength	Refractive Index (Measured)	Refractive Index (Reference) [31]
Cyclohexane	1290 nm	1.4175	1.4177
	1560 nm	1.4170	1.4170
Benzene	1290 nm	1.4805	1.4781
	1560 nm	1.483	1.4769
Toluene	1290 nm	1.4819	1.4791
	1560 nm	1.4726	1.4777
